# Coherence of evidence from systematic reviews as a basis for evidence strength - a case study in support of an epistemological proposition

**DOI:** 10.1186/1756-0500-5-26

**Published:** 2012-01-12

**Authors:** Steffen Mickenautsch

**Affiliations:** 1 SYSTEM Initiative/Department of Community Dentistry, Faculty of Health Sciences, University of the Witwatersrand, 7 York Rd., Parktown/Johannesburg 2193, South Africa

## Abstract

**Background:**

This article aims to offer, on the basis of Coherence theory, the epistemological proposition that mutually supportive evidence from multiple systematic reviews may successfully refute radical, philosophical scepticism.

**Methods:**

A case study including seven systematic reviews is presented with the objective of refuting radical philosophical scepticism towards the belief that glass-ionomer cements (GIC) are beneficial in tooth caries therapy. The case study illustrates how principles of logical and empirical coherence may be applied as evidence in support of specific beliefs in healthcare.

**Results:**

The results show that radical scepticism may epistemologically be refuted on the basis of logical and empirical coherence. For success, several systematic reviews covering interconnected beliefs are needed. In praxis, these systematic reviews would also need to be of high quality and its conclusions based on reviewed high quality trials.

**Conclusions:**

A refutation of radical philosophical scepticism to clinical evidence may be achieved, if and only if such evidence is based on the logical and empirical coherence of multiple systematic review results. Practical application also requires focus on the quality of the systematic reviews and reviewed trials.

## Introduction

Epistemology is described as the branch of philosophy that concerns itself with questions regarding human knowledge [1]. One particular epistemological question relates to the *Object - Subject *distinction and asks whether the objective world is subjectively knowable at all. It has been proposed that the real (objective) world and our (subjective) perception of the real world are not the same. Descartes (1641) argued that all that we can know of the real world is tainted by our senses and abilities of understanding [2]. Kant (1998 [1781]) distinguished between the unknowable *Ding an sich *(German: The thing in itself) and the knowable *Erscheinung *(German: Phenomenon) [3]. Postmodernism contends that the notion of reality is an illusion [4]. Quine (1964) described physical objects as mere cultural posits [5], and cognitive psychology and neuroscience have presented evidence that sense experience, the 'bedrock of empirical knowledge', is actively edited by human perception [4]. Such 'editing' renders sense experience subjective to the particular observer [6].

All these examples [2-6] share the common concept that some form of bias determines subjective perception concerning real world events. Bias, also called systematic error, manifests in various forms and number, and constitutes any factor in the knowledge acquisition process that systematically diverts its outcomes from true values [7]. The concept of knowledge has been defined as true and justified belief [1]. Systematic error, therefore, limits the trueness and thus justifiability of such belief (thus its value to be considered as knowledge) and so widens the gap between the real (objective) world and our (subjective) perception of it. In order to limit the influence of some forms of systematic errors in the field of healthcare, methodological interventions for clinical trials, such as randomisation, blinding and intention-to-treat analysis, are proposed [8] and have been shown to be effective [9,10]. Clinical trials that employ such methodological interventions are identified and synthesized through systematic reviews. Systematic reviews present: (i) a synthesis of the acquired knowledge regarding one particular clinical question derived from all relevant studies that are identifiable at one point in time, (ii) identify the level of validity and the subsequent potential systematic error risk associated with the acquired knowledge and (iii) provide recommendations for improving any identified shortcoming related to internal validity, for further research. Owing to continued further research, systematic reviews also provide continued updates of their synthesis [10]. For this reason, in most cases systematic reviews provide the most objective knowledge possible about real world events; such as answers to clinical questions concerning causalities, with the least possible systematic error.

However, the described distinction between the real (objective) world and (subjective) perception of the real world [2-6] has also given ground to radical, philosophical scepticism about whether it is possible to gain knowledge of real (objective) world events, at all [1]. Radical, philosophical scepticism does not just question current standards of evidence: it completely denies that any causality existing independently from our perception in the real world is knowable at all; regardless of the availability and strength of evidence, e.g. as presented in systematic reviews that supports knowledge of such causality. By disregarding such evidence, radical, philosophical scepticism denies any justification for or against any perception of objective knowledge. Among the main modes of argument utilized in radical, philosophical scepticism are the five modes of Agrippa [1,11]. These modes of argument state: (i) for each thesis there is a possible antithesis but without the option of Fichte's synthesis through thesis and antithesis [12] - because: (ii) each point of view is relative to each particular claimant and thus cannot hold general truth; because: (iii) proof of any point of view or thesis is not possible, as every proof, evidence or justification requires for its own proof, evidence or justification, thus entering into an infinite regress of proof that is incapable of proving anything; (iv) to end such infinite regress of proof by refusing further proof shows lack of proof; and (v) to reason in circles by referring back to already stated proof also shows lack of proof [11].

In accordance with the Agrippean argument, evidence elicited from single systematic reviews as proof in clinical practice is vulnerable to radical scepticism. An example is the argument that population-based research (research conducted, e.g. through randomised control trials (RCT) and subsequently appraised through systematic reviews) cannot be applicable to the treatment of individual patients [13]. The validity of population-based results, so it is argued, may only be applied to the population as such but remains irrelevant to the single patient, as individual patient data and a population average are never the same [13]. Such criticism denies the ability of systematic review evidence to describe objective causality. It employs the Agrippean argument that: (i) like any other thesis, the thesis of objective evidence by systematic reviews is contradictable; (ii) the thesis of objective evidence by systematic reviews (the population average) is relative and thus cannot be valid for the individual patient because the general validity of such thesis cannot be proven (see Agrippean modes of argument iii - v).

The aim of this article is to offer the epistemological proposition that, with reference to the Coherence theory and illustrated by one case study, evidence from multiple systematic reviews, if mutually supportive (= logically and empirically coherent) may be able in principle and on philosophical ground to terminate the infinite regress of proof and thus refute the Agrippean argument against it. This article further aims to highlight some aspects concerning the translation of such proposition from philosophical grounds into health care praxis.

## Coherence theory

The key to refuting the Agrippean argument is to terminate the infinite regression of proof. The radical sceptic rejects refusal of further proof and circular reference to proof, preventing formation of a successful refutation. A possible epistemological solution to this problem is to assert that not just one single belief but many, are expressed in a thesis. Such beliefs do not exist independently from each other but form a system or web of mutually supporting beliefs that justify each other. This line of argument against radical scepticism has been called the Coherence theory [14-16]. The Coherence theory states that beliefs are justified if they are systematically interconnected and provide mutual proof for each other [1]. Such mutual proofs do not constitute mere circularity but logical coherence, thus terminating the need for infinite regression of proof. According to Coherence theory in epistemology, justification of proof of a belief depends on how well it fits into the web of beliefs, thus stating that individual beliefs are justified by virtue of belonging to a coherent view. This argument has been criticized on the grounds that it allows any form of belief, no matter how unlikely or likely; to be considered as justified as long as it is surrounded by suitable supporting beliefs [1]. Even if such supporting beliefs are as unlikely as the main belief, it may still form a coherent system. Against such background, the Coherence theory is unable to make a clear distinction between aspects of the real world (causality) and delusions. However, it has been argued that the Coherence theory is not a theory of truth but a theory of justification [1]. The distinction between a coherent real world system and a coherent system of delusion can be made only on the basis of its relation to evidence. Such evidence has to be empirical: facts about the real world ascertained from observation and experiment. For a coherent system to be proof of truth, it not only needs to be: (i) logically coherent in terms of its interconnecting beliefs, but also (ii) coherent in terms of the empirical evidence on which all interconnecting beliefs are based (empirical coherence).

## Case study

In order to show how the above principles of Coherence theory may be applied in support of specific beliefs in healthcare, a case study including seven systematic reviews [17-23], all co-written and published by the author, was conducted. These systematic reviews related to various aspects of the clinical use of glass-ionomer cements (GIC) and dental caries. The format of a case study was chosen in order to explore without high methodological restrictions the potential usefulness of Coherence theory in this context and to contribute new insights for discussion and further study. As Coherence theory is rooted in philosophical/epistemological thinking, this article limits its line of argument mainly on philosophical/epistemological grounds. However, some practical implications, relevant to the field of health care are also discussed.

The specific objective of this case study was to illustrate, as example, the refutation of radical scepticism towards the belief that GICs are beneficial in tooth caries therapy on the basis of logical and empirical coherence, and included the following steps:

(i) Conducting and publication of systematic review articles addressing different review questions concerning the main belief regarding clinical benefits of GICs in tooth caries therapy;

(ii) Extraction of 'web'components, separated by common categories, that are contained in the systematic reviews;

(iii) Construction of a 'web-of-beliefs' frame from the extracted components;

(iv) Setting of conditions that need to be in place in the constructed 'web-of-beliefs' in order for it to be considered coherent;

(v) Discussion about whether the specific information provided by the systematic reviews fulfils the conditions for (a) logical coherence and (b) empirical coherence.

### Systematic review sample

The format of systematic reviews of trials was chosen above other forms of scientific investigation (i.e. randomised control trials, observational studies, clinical case reports or narrative reviews/expert opinion), as these are considered to provide the most comprehensive answers to clinical questions, with the least possible systematic error [10]. All systematic reviews were of quantitative (as opposed to qualitative) nature. Some included meta-analyses for precision of results. The systematic reviews were undertaken in order to answer separate clinical review questions, but all with relevance to the main belief that GIC is beneficial in caries therapy. All systematic reviews were published in MEDLINE- listed peer-reviewed dental journals between September 2009 and February 2011. The language of publication was English.

Of the seven systematic reviews, three included conventional GIC (C-GIC) [17,18,22] and three, resin-modified GIC (RM-GIC) [19,20,23] as the test material. One systematic review investigated both versions of GIC, in comparison [21]. Two systematic reviews investigated the fissure sealant performance of GIC [17,19]. The other five investigated its performance as a material for tooth restorations [18,20-23]. The investigated outcome measures of the seven systematic reviews were tooth remineralisation [20], absence of tooth caries [17-19,21,23] and long-time survival of tooth restorations [22].

The strength of systematic review evidence relies on (i) the quality of trials reviewed and (ii) the quality of the systematic review itself. The AMSTAR (Assessment of Multiple SysTemAtic Reviews) tool [24-26] was used for critical self-assessment of the quality of the systematic review articles. The results are shown in Table 1. On the basis of this assessment, the AMSTAR score for three systematic reviews was 9 [19,21,23], three systematic reviews scored 8 [18,20,22] and one scored 7 [17]. An AMSTAR score between 8 and 11 has been suggested to indicate a high level of systematic review quality and a score between 4 and 7, a medium one [27]. According to such interpretation, six of the seven systematic reviews were of high quality and one of medium quality. However, the quality of systematic review results can only be as good as the quality of its reviewed trials.

**Table 1:** Result of critical self-assessment of systematic review quality (AMSTAR)

AMSTAR components	**Yengopal et al., 2009 **[17]	**Yengopal and Mickenautsch, 2010 **[19]	**Mickenautsch et al., 2010 **[21]	**Mickenautsch et al., 2010 **[22]	**Mickenautsch et al., 2009 **[18]	**Mickenautsch and Yengopal, 2010 **[20]	**Yengopal and Mickenautsch, 2011 **[23]
(1) **Was an 'a priori' design provided? **The research question and inclusion criteria should be established before the conduct of the review.	X	X	X	X	X	X	X

(2) **Was there duplicate study selection and data extraction? **There should be at least two independent data extractors and a consensus procedure for disagreements should be in place.	X	X	X	X	X	X	X

(3) **Was a comprehensive literature search performed? **At least two electronic sources should be searched. The report must include years and databases used (e.g. Central, EMBASE, and MEDLINE). Key words and/or MeSH terms must be stated and where feasible the search strategy should be provided. All searches should be supplemented by consulting current contents, textbooks, specialized registers, or experts in the particular field of study, and by reviewing the references in the studies found.	X	X	X	X	X	X	X

(4) **Was the status of publication (i.e. grey literature) used as an inclusion criterion? **The authors should state that they searched for reports regardless of their publication type. The authors should state whether or not they excluded any reports (from the systematic review), based on their publication status, language etc.	X	X	X		X		

(5) **Was a list of studies (included and excluded) provided? **A list of included and excluded studies should be provided.	X	X	X	X	X	X	X

(6) **Were the characteristics of the included studies provided? **In an aggregated form such as table, data from the original studies should be provided on the participants, interventions and outcomes. The ranges of characteristics in all studies analysed e.g. age, race, sex, relevant socio-economic data, disease status, severity, or other diseases should be reported.	X	X	X	X	X	X	X

(7) **Was the scientific quality of the included studies assessed and documented? **'A priori' methods of assessment should be provided (e.g. for effectiveness studies if the author(s) chose to include only randomised, double-blind, placebo controlled studies or allocation concealment as inclusion criteria); or other types of studies alternative items will be relevant.	X	X	X	X	X	X	X

(8) **Was the scientific quality of the included studies used appropriately in formulating conclusions? **The result of the methodological rigor and scientific quality should be considered in the analysis and the conclusions of the review and explicitly stated in formulating recommendations.		X	X	X		X	X

(9) **Were the methods used to combine the findings of the studies appropriate? **For the pooled results, a test should be done to ensure the studies were combinable, to assess their homogeneity (i.e. Chi-squared test for homogeneity, I^2^). If heterogeneity exists a random effects model should be used and/or the clinical appropriateness of combining should be taken into consideration (i.e. is it sensible to combine?).		X	X	X	X	X	X

(10) **Was the likelihood of publication bias assessed? **An assessment of publication bias should include a combination of graphical aids (e.g. funnel plot, other available test) and/or statistical tests (e.g. Egger regression test).							X

(11) **Was the conflict of interest stated? **Potential source of support should be clearly acknowledged in both the systematic review and the included studies.							

The use of the AMSTAR tool does not establish whether systematic reviews judged the quality of trials according to whether or not bias was successfully prevented. Selection bias, besides e.g. detection/performance-, attrition- or publication bias, is one of the most important types of bias in clinical trials and effectively diverts trial results away from the truth, even in absence of any other types of bias risk [8]. Therefore, selection bias due to lack of adequate concealment of the random sequence allocation was assessed in all reviewed trials. Allocation, using for example central randomisation, was considered to be adequate concealment. The result showed that none of the 47 trials accepted for review included adequate allocation concealment in their methodology.

### Web-of-beliefs and components

All beliefs investigated by the seven systematic reviews: belief related to GICs remineralising effect (Belief 1), caries-preventive effect (Belief 2), restoration survival (Belief 3), are mutual to the main belief regarding GIC's benefit in caries therapy. All systematic reviews contained the component categories: Test material (T_n_); Control material (C_n_); Treatment type (TN_n_) and Outcome measure (OM_n_). Details about the specific components per category are shown in Table 2. These various component categories interconnect by virtue of the methodology employed in the systematic reviews, thus forming strings, such as:

**Table 2:** Case study: web of beliefs components

Category	Component	Symbol
Test material	Resin-modified GIC	T_1_
	
	Conventional GIC	T_2_

Control material	Resin	C_1_
	
	Composite resin	C_2_
	
	Amalgam	C_3_
	
	Other	C_4_

Treatment type	Tooth restoration	TN_1_
	
	Tooth pits and fissure sealant	TN_2_

Outcome measure	Tooth remineralisation	OM_1_
	
	Absence of carious tooth lesions	OM_2_
	
	Tooth restoration survival	OM_3_

*GIC *glass-ionomer cement

$$\begin{array}{c} \left\{Test\;material-control\;material-investigated\;treatment\;type-outcome\;measure\right\}\text{or}\\ \;\;\;\;\;\;\;\;\;\;\;\;\;\;\;\;\;\;\{T_{n}-C_{n}-TN_{n}-OM_{n}\text{\}} \end{array}$$

**Figure 1:**
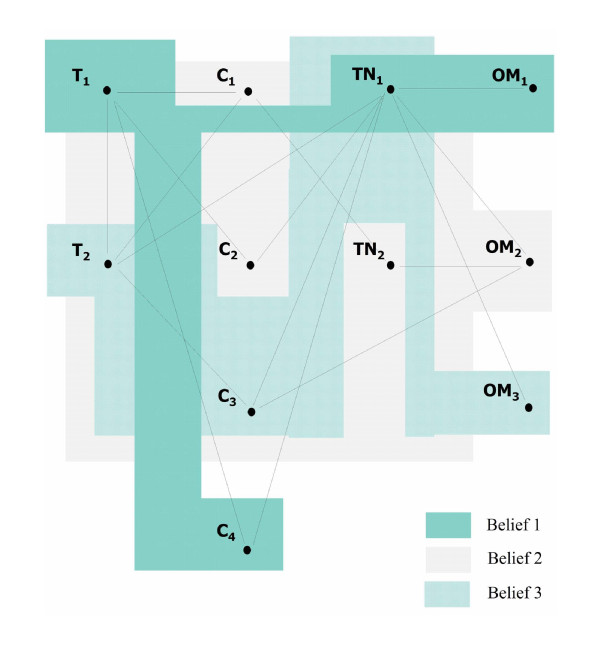
**Case study: graphical illustration of web of beliefs frame**. See Table 2 for explanation of symbols.

Through the various components present in each systematic review per category, various different strings may thus be generated (Table 3). The various beliefs are expressed and tested along these constructed methodological strings. As each string makes use of at least one of the various components, these strings can be graphically merged and thus illustrate the outline structure of a 'web-of-beliefs' frame, relevant to the overall topic as to whether GIC is beneficial in caries therapy (Figure 1). The illustration of such frame in the form of a graph gives an overview of how the various beliefs are connected and form into a 'web'. Figure 1 also serves as a map to graphically discern the interconnections between the various requirements of coherence.

**Table 3:** Case study: constructed methodological strings per systematic review

Beliefs related to:	Systematic review	Methodological string
Belief 1: Remineralising effect of GIC	Mickenautsch and Yengopal, 2010 [20]	*{T_1 _- C_4 _- TN_1 _- OM_1_}*

Belief 2: Caries-preventive effect of GIC	Yengopal et al., 2009 [17]	*{T_2 _- C_1 _- TN_2 _- OM_2_}*
	
	Yengopal and Mickenautsch, 2010 [19]	*{T_1 _- C_1 _- TN_2 _- OM_2_}*
	
	Mickenautsch et al., 2010 [21]	*{T_1 _- T_2 _- TN_1 _- OM_2_}*
	
	Mickenautsch et al., 2009 [18]	*{T_2 _- C_3 _- TN_1 _- OM_2_}*
	
	Yengopal and Mickenautsch, 2011 [23]	*{T_1 _- C_2 _- TN_1 _- OM_2_}*

Belief 3: GIC restoration survival	Mickenautsch et al., 2010 [22]	*{T_2 _- C_3 _- TN_1 _- OM_3_}*

### Requirements for coherence

Whether this web-of-beliefs is indeed 'coherent' depends on how well its underlying concepts are logically coherent, as well as on the empirical coherence of its underlying clinical evidence.

Within the framework of this case study, logical coherence was based on the tautologies that:

(i) Tooth remineralisation (OM1) is conceptually closer related to absence of carious tooth lesions (OM2) than to lesion presence;

(ii) Absence of carious tooth lesions (OM2) on restoration margins is conceptually closer related to tooth restoration survival (OM3) than to failure.

In biological/clinical terms, these tautologies are oversimplified, as the process of the caries disease is rather complex. However, within the philosophical context of this case study, these tautologies mean that the higher the tooth remineralisation effect, the less the occurrence of carious tooth lesions is expected to be and the less the occurrence of lesions on restoration margins, the higher the survival of tooth restorations is expected to be.

Coherence of clinical evidence (Empirical coherence) will mean that the following conditions are fulfilled, within the framework of this case study:

(i) The remineralisation effect (OM_1_) associated with test materials (T_n_) is at least not lower than with control materials (C_n_), regardless of the type of treatment (TN_n_);

(ii) Absence of carious tooth lesions (OM_2_) associated with test materials (T_n_) is at least not lower than with control materials (C_n_), regardless of the type of treatment (TN_n_);

(iii) Tooth restoration survival (OM_3_) associated with test materials (T_n_) is at least not lower than with control materials (C_n_), for tooth restoration as treatment type (TN_1_);

(iv) There are no differences in the clinical outcomes between the different versions of the test materials (T_1 and 2_) regardless of type of treatment (TN_n_) and outcome measure (OM_n_);

(v) There is no difference between the clinical outcomes of the same version of the test material (T_1 or 2_) in comparison to different control materials (C_n_), (under condition of same type of treatment (TN_n_) and outcome measure (OM_n_);

(vi) There is no difference between the clinical outcomes of the different version of test material (T_n_) in comparison to the same control material (C_1,2,3 or 4_) (under condition of same type of treatment (TN_n_) and outcome measure (OM_n_).

The information provided by the systematic reviews were discussed in relation to these conditions, to establish whether it complied with the requirements for coherence:

### Logical coherence

In order to fulfil the condition of logical coherence the tautologies that: (i) the higher the tooth remineralisation effect, the less the occurrence of carious tooth lesions and (ii) the less the occurrence of carious tooth lesions on restoration margins, the higher will be the occurrence of the survival of tooth restorations, were adopted. Tautology is a philosophical term that describes a statement that is true in every circumstance, thus forming a necessary truth [28]. This necessary truth is circumscribed in the commonly accepted definition of carious tooth lesions [29] and its occurrence on restorative margins as a common cause of tooth restoration failure [30].

### Empirical coherence

The evidence provided by the seven systematic reviews complies with all set conditions for coherence of evidence (Table 4). No non-compliance that would be in contradiction of coherence within the established web-of-beliefs has been identified: Remineralisation in association with GIC has not been found lower than with the control [20]. Such remineralising effect supports the belief that GIC is not associated with a higher caries risk (= logical coherence tautology) and that belief has been confirmed [17-19,23]. Such caries resistance in turn supports the belief that the survival rate of GIC-based (ART) tooth restorations is at least not lower than that of the control (= logical coherence tautology). That belief was shown to be correct [22]. In addition, the different versions of GIC do not give different clinical results, because they both contain the same active ingredient [17,19,21] and the anti-cariogenic efficacy of GIC was shown, even when compared against those of different control materials [17-19,23]. Against this background and within the set web-of-beliefs, empirical coherence appears to be confirmed.

**Table 4:** Case study: compliance of systematic review evidence with conditions for coherence of evidence

Systematic review	Review conclusion	Evidence in regard to conditions for coherence of evidence					
		
		(i)	(ii)	(iii)	(iv)	(v)	(vi)
Yengopal et al., 2009 [17]	... indicating no difference in the caries-preventive effect of GIC and resin-based fissure sealant material		2			5a	6

Yengopal and Mickenautsch, 2010 [19]	... found no conclusive evidence that either material was superior to the other in preventing dental caries		2			5b	6

Mickenautsch et al., 2010 [21]	... indicating no difference in the caries preventive effect between both types of materials				4		

Mickenautsch et al., 2010 [22]	In the permanent dentition the longevity of ART is equal to or greater than that of equivalent amalgam restorations for up to 6.3 years and is site-dependent. No difference was observed in primary teeth.			3			

Mickenautsch et al., 2009 [18]	Carious lesions at margins of single-surface GIC restorations are less common than with amalgam fillings after 6 years in permanent teeth. No difference was observed in primary teeth.		2			5a	

Mickenautsch and Yengopal, 2010 [20]	The evidence suggests that RM-GIC is associated with a higher reduction of demineralization in adjacent hard tooth tissue than that of composite resin without fluoride. No difference was found when RM-GIC was compared with fluoride-containing composite.	1					

Yengopal and Mickenautsch, 2011 [23]	The overall results showed no difference between the materials or indicated that RM-GIC has a superior caries-preventive effect.		2			5b	

Evidence	Compliance/non-compliance with conditions for coherence of evidence						

1	Compliance. No lower remineralisation than that of control was observed, as per condition (i).						

2	Compliance. No lower absence of caries than that of control was observed, as per condition (ii).						

3	Compliance. No lower survival rate was observed, as per condition (iii).						

4	Compliance. No difference was observed, as per condition (iv).						

5a	Compliance. No difference was observed for RM-GIC, as per condition (v).						

5b	Compliance. No difference was observed for GIC, as per condition (v).						

6	Compliance. No difference was observed, as per condition (vi).						

*GIC *glass-ionomer cement; *ART *tooth restoration with GIC; *RM-GIC *resin-modified GIC.

### Further aspects of evidence

In line with the Coherence theory, the logical and empirical coherence presented in this case study may be regarded as sufficient to terminate the infinite regression of proof imposed by radical scepticism. This means that radical philosophical scepticism towards the clinical benefit of GIC regarding caries may be successfully refuted in epistemological terms. However, it is important to note that such epistemological proposition can only be translated successfully into practice if and only if no general scepticism concerning the validity of the underlying empirical evidence arises. In the presented case study, empirical evidence is provided by seven systematic reviews. Systematic reviews are regarded as providing the highest form of evidence to healthcare questions [10]. Nonetheless, systematic reviews do not by virtue of their nature provide high empirical evidence. Rather, such evidence can be regarded as valid only when the systematic reviews and the trials reviewed are of high quality. The AMSTAR tool has been proposed as one method for measuring the quality of systematic reviews. Critical self-assessment by use of the AMSTAR tool showed a general high quality in the systematic reviews included in the case study (Table 1). However, even if empirical evidence is provided by high-quality systematic reviews, this evidence can only be as valid as the evidence provided by the clinical trials reviewed. Assessment of all 47 trials found reason for doubt (= lack of reporting adequate random allocation concealment) of evidence validity, owing to the high risk of selection bias. It has been emphasized that selection bias can be successfully prevented only if the allocation sequence remains truly random and free from potential interference throughout the trial [31,32]. Thus, it is important that trials should include an effective process for concealing the random allocation sequence and that by the end of each trial this process has indeed prevented direct observation and prediction of the random sequence allocation [31,32]. Egger et al., 2003 reported a treatment effect overestimation of between 21% and 54% due to selection bias, solely caused by lack of allocation concealment during the randomisation process [9]. The seriousness of such overestimation becomes clear under consideration that in a condition of a 50% overestimation, the actual result for a test treatment would be a 20% higher relative risk (RR 1.20) than for the control, while the trial report would claim a 20% lower relative risk (RR 0.80). Thus, in this example, the true result of the trial would constitute the complete opposite of the reported trial result. Such high percentages of over-estimation due to bias may therefore lead to situations where ineffective treatment procedures are presented as effective. For this reason, all trial results identified in the seven systematic reviews need to be interpreted with caution.

## Conclusion

Radical, philosophical scepticism towards the benefit of clinical procedures in healthcare may epistemologically be refuted on the basis of logical and empirical coherence. Such coherence indicates causality in the real (objective) world - in this example GIC related to caries-therapeutic benefit - that exists independently of (subjective) perceptions or scepticism. Empirical coherence is provided from clinical evidence. Such evidence is acquired through clinical trials and subsequently synthesised by (quantitative) systematic reviews. A single systematic review covering one topic may be insufficient for refuting the Agrippean argument. For this, several systematic reviews covering interconnected beliefs may be needed. It is stressed that a successful application of such epistemological proposition into practice regarding refutation of radical, philosophical scepticism towards the benefit of a particular clinical procedure is possible only if these systematic reviews are of high quality and the quality of reviewed clinical trials is high enough to withstand general scepticism concerning trial validity.

## Authors' contributions

SM developed the concept and outline and wrote this paper.

## Competing interests

The author contributes to the conduct and publication of systematic reviews concerned with topics related to Minimum Intervention (MI) in dentistry.
